# Quantitative assessment of the effectiveness of joint measures led by Fangcang shelter hospitals in response to COVID-19 epidemic in Wuhan, China

**DOI:** 10.1186/s12879-021-06165-w

**Published:** 2021-07-01

**Authors:** Hui Jiang, Pengfei Song, Siyi Wang, Shuangshuang Yin, Jinfeng Yin, Chendi Zhu, Chao Cai, Wangli Xu, Weimin Li

**Affiliations:** 1 Beijing Chest Hospital, Capital Medical University, Beijing, 101149 China; 2 Beijing Tuberculosis and Thoracic Tumor Research Institute, Beijing, 101149 China; 3 School of Mathematics and Statistics, Xi’an Jiaotong University, Xi’an, 710049 ShaanXi China; 4 Center for Applied Statistics, School of Statistics, Renmin University of China, Beijing, 100872 China; 5 Beijing Youan Hospital, Capital Medical University, Beijing, 100069 China; 6 Beijing Municipal Key Laboratory of Clinical Epidemiology, School of Public Health, Capital Medical University, Beijing, 100069 China

**Keywords:** COVID-19, Fangcang shelter hospital, Quantitative assessment, Effectiveness

## Abstract

**Objective:**

To quantitatively evaluate the effectiveness of Fangcang shelter hospitals, designated hospitals, and the time interval from illness onset to diagnosis toward the prevention and control of the COVID-19 epidemic.

**Methods:**

We used SEIAR and SEIA-CQFH warehouse models to simulate the two-period epidemic in Wuhan and calculate the time dependent basic reproduction numbers (BRNs) of symptomatic infected individuals, asymptomatic infected individuals, exposed individuals, and community-isolated infected individuals. Scenarios that varied in terms of the maximum numbers of open beds in Fangcang shelter hospitals and designated hospitals, and the time intervals from illness onset to hospitals visit and diagnosis were considered to quantitatively assess the optimal measures.

**Results:**

The BRN decreased from 4.50 on Jan 22, 2020 to 0.18 on March 18, 2020. Without Fangcang shelter hospitals, the cumulative numbers of cases and deaths would increase by 18.58 and 51.73%, respectively. If the number of beds in the designated hospitals decreased by 1/2 and 1/4, the number of cumulative cases would increase by 178.04 and 92.1%, respectively. If the time interval from illness onset to hospital visit was 4 days, the number of cumulative cases and deaths would increase by 2.79 and 6.19%, respectively. If Fangcang shelter hospitals were not established, the number of beds in designated hospitals reduced 1/4, and the time interval from visiting hospitals to diagnosis became 4 days, the cumulative number of cases would increase by 268.97%.

**Conclusion:**

The declining BRNs indicate the high effectiveness of the joint measures. The joint measures led by Fangcang shelter hospitals are crucial and need to be rolled out globally, especially when medical resources are limited.

**Supplementary Information:**

The online version contains supplementary material available at 10.1186/s12879-021-06165-w.

## Background

In December 2019, the coronavirus disease 2019 (COVID-19) outbreak occurred in Wuhan, China. Subsequently, it occurred in many countries around the world, after which WHO announced COVID-19 as a global pandemic on 11 March, 2020 [1]. In the early period of the epidemic in Wuhan, thousands of cases rushed to hospitals, pressurizing the city’s medical system [2]. To manage the serious situation of COVID-19, strategies of joint prevention and control through a triage were adopted. Mild/moderate cases and close contacts were isolated in community and quarantine points, severe and critical cases were admitted to designated hospitals.

By implementing multiple prevention and control measures, such as community isolation, quarantine point isolation, designated hospitals, and employing new diagnostic and intervention techniques, rational allocation of medical resources and services was guaranteed during the COVID-19 epidemic. However, there was still a huge severity of treatment pressure, such as lack of beds and medical resources. To relieve pressure, 86 designated hospitals providing approximately 24,000 beds were rebuilt and re-established [3], and a total of 344 national medical teams with a medical staff of 42,322 were dispatched [4]. In addition, from February 05, Wuhan successively established and opened 16 Fangcang shelter hospitals, providing about 13,000 beds to admit mild/moderate cases symptoms. The implementation of multi-stage joint measures and multi-sectoral division of labor and cooperation have played an important role in the response to COVID-19. However, although the function of Fangcang shelter hospitals has been defined in previous studies [2], the role of these hospitals in joint measures has not been quantitatively evaluated. Further, no study has quantitatively evaluated the role of joint measures such as establishing Fangcang shelter hospitals, expanding designated hospitals, and shortening the time interval from illness onset to diagnosis in response to the COVID-19 epidemic.

In this study, we evaluated the effectiveness of joint measures led by Fangcang shelter hospitals after the closure of Wuhan on January 23, 2020 [3]. In addition, to include the asymptomatic infected individuals, we extended the classic suspected-exposed-infected-recovered (SEIR) transmission model to the suspected-exposed-symptomatic infected-asymptomatic infected-recovered (SEIAR) model for describing the epidemiological characteristics. Four additional compartments (community isolation [C], quarantine point isolation [Q], Fangcang shelter hospitals [F], and designated hospitals [H]) were added to quantitatively assess Fangcang shelter hospitals for the COVID-19 epidemic in Wuhan from January 23, 2020 to March 18, 2020.

## Methods

### Data sources and collection

From the National Health Commission of the People’s Republic of China and WHO, we collected the numbers of newly confirmed cases, cumulative confirmed cases, and deaths of COVID-19 in Wuhan from January 23 to March 18, 2020 [1, 5]. The data of the maximum numbers of open beds in Fangcang shelter hospitals (Supplementary Figure 1A), designated hospitals (Supplementary Figure 1B), and quarantine points were obtained from Wuhan Municipal Health Commission [3].

### Data analysis

#### SEIAR and SEIAR-CQFH model to stimulate two-period epidemic in Wuhan

**Fig. 1:**
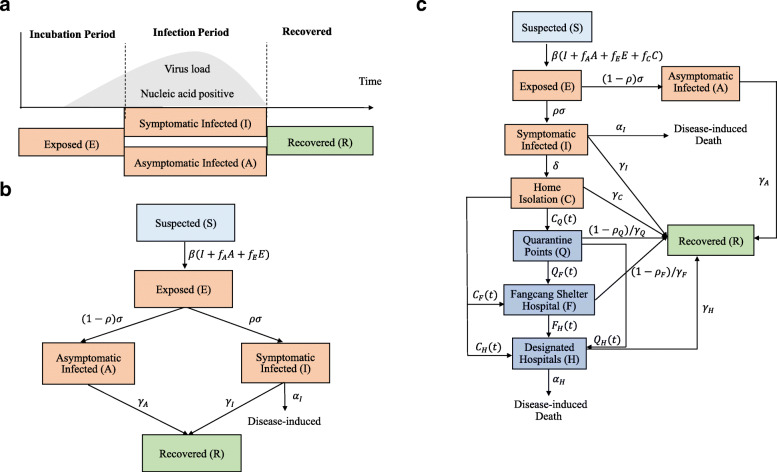
Descriptions of the first and second periods by SEIAR and SEIAR-CQFH models. Panel **a**: Epidemiological descriptions of exposed (**e**), symptomatic infected (I) and asymptomatic infected (A) individuals. Panel **b**: Descriptions of the first period (December 7 to January 22) by SEIAR model. Panel **c**: Descriptions of the second period (January 23 to March 18) by SEIAR-CQFH model

Based on the date of Wuhan lockdown, we divided the Wuhan epidemic into two periods: December 7, 2019 to 2020 January 22 and January 23, 2020 to March 18, 2020; SEIAR and SEIA-CQFH warehouse models were employed to simulate these two periods of Wuhan epidemic, respectively. For the first period epidemic, we extended the basic SEIR model to the SEIAR model by enrolling asymptomatic infected individuals as follows (Fig. 1a-b) [6].

$$ \left\{\begin{array}{l}\frac{\mathrm{d}S}{\mathrm{d}\mathrm{t}}=-{\beta}_0\left(I+{f}_AA+{f}_EE\right)\frac{S}{N},\\ {}\frac{\mathrm{d}E}{\mathrm{d}\mathrm{t}}={\beta}_0\left(I+{f}_AA+{f}_EE\right)\frac{S}{N}-\sigma E,\\ {}\frac{\mathrm{d}I}{\mathrm{d}\mathrm{t}}=\rho \sigma E-{\gamma}_II-{\alpha}_II,\\ {}\frac{\mathrm{d}A}{\mathrm{d}\mathrm{t}}=\left(1-\rho \right)\sigma E-{\gamma}_AA,\\ {}\frac{\mathrm{d}R}{\mathrm{d}\mathrm{t}}={\gamma}_II+{\gamma}_AA,\end{array}\right. $$

where *S*(*t*), *E*(*t*), *I*(*t*), *A*(*t*), *R*(*t*) and *N* = *S*(*t*) + *E*(*t*) + *I*(*t*) + *A*(*t*) + *R*(*t*) are the number of susceptible, exposed, symptomatic infected, asymptomatic infected, recovered individuals and the total population of Wuhan at time *t*, respectively. The functions *S*(*t*), *E*(*t*), *I*(*t*), *A*(*t*), *R*(*t*) that are dependent on *t* are simply denoted as *S*, *E*, *I*, *A*, *R* in the SEIAR model.

For the second period, we extended the SEIAR model to enroll clinically diagnosed cases in community isolation (C), quarantine point isolation (Q), Fangcang shelter hospitals (F) and designated hospitals (H). The SEIAR-CQFH model is described as follows (Fig. 1c):

$$ \left\{\begin{array}{l}\frac{\mathrm{d}S}{\mathrm{d}\mathrm{t}}=-\beta (t)\left(I+{f}_AA+{f}_EE+{f}_CC\right)\frac{S}{N},\\ {}\frac{\mathrm{d}E}{\mathrm{d}\mathrm{t}}=\beta (t)\left(I+{f}_AA+{f}_EE+{f}_CC\right)\frac{S}{N}-\sigma E,\\ {}\frac{\mathrm{d}I}{\mathrm{d}\mathrm{t}}=\rho \sigma E-{\gamma}_II-{\alpha}_II-\delta I,\\ {}\frac{\mathrm{d}A}{\mathrm{d}\mathrm{t}}=\left(1-\rho \right)\sigma E-{\gamma}_AA,\\ {}\frac{\mathrm{d}C}{\mathrm{d}\mathrm{t}}=\delta I-{\gamma}_CC-{C}_H(t)-{C}_Q(t)-{C}_F(t),\\ {}\frac{\mathrm{d}Q}{\mathrm{d}\mathrm{t}}={C}_Q(t)-\left(1-{\rho}_Q\right){\gamma}_QQ-{Q}_F(t)-{Q}_H(t),\\ {}\frac{\mathrm{d}F}{\mathrm{d}\mathrm{t}}={Q}_F(t)+{C}_F(t)-\left(1-{\rho}_F\right){\gamma}_FF-{F}_H(t),\\ {}\frac{\mathrm{d}H}{\mathrm{d}\mathrm{t}}={C}_H(t)+{Q}_H(t)+{F}_H(t)-{\gamma}_HH-{\alpha}_HH,\\ {}\frac{\mathrm{d}R}{\mathrm{d}\mathrm{t}}={\gamma}_II+{\gamma}_AA+{\gamma}_CC+\left(1-{\rho}_Q\right){\gamma}_QQ+\left(1-{\rho}_F\right){\gamma}_FF+{\gamma}_HH,\end{array}\right. $$

where *S*(*t*), *E*(*t*), *I*(*t*), *A*(*t*), *R*(*t*), *N* have the same definitions as those in the SEIAR model, and *C*(*t*), *Q*(*t*), *F*(*t*), *H*(*t*) are the number of community isolation, quarantine point isolation, Fangcang shelter hospitals, and designated hospitals at time *t*, respectively. More details about the model parameters and function settings are presented in the supplementary.

Some parameters were determined from existing references (refer to Table 1 for details), and seven unknown parameters (*β*_0_, *β*_*end*_, *r*, *α*_*I*_, *α*_*H*_, *δ*_*cq*_, *θ*) were estimated by the nonlinear least-squares (NLES) method based on newly confirmed, cumulative confirmed, and cumulative COVID-19 death cases in Wuhan from January 23 to March 18. The confidence intervals of the parameters were calculated using the stochastic simulation method (Table 1).

**Table 1:** Parameters used in the SEIAR and SEIAR-CQFH models

Parameter	Implication	Value	Sources
*β*_0_	The transmission rate of symptomatic infectious before Jan 23	0.43 (95%CI: 0.428–0.431)	Estimated
*β*_*end*_	The minimum transmission rate of symptomatic infectious under interventions after Jan 23	0.05 (95%CI: 0.049–0.051)	Estimated
*r*	Exponential decreasing rate of transmission rate of symptomatic infectious under interventions after Jan 23	0.072 (95%CI: 0.0715–0.0728)	Estimated
*f*_*E*_	The fitted transmission rate with respect to exposed individuals	1/3	Ref [7]
*f*_*A*_	The fitted transmission rate with respect to asymptomatic infectious	1/3	Ref [7]
*f*_*C*_	The fitted transmission rate with respect to home isolated symptomatic infectious	1	Assumed
1/*σ*	Incubation period	5.2	Ref [8–10]
*ρ*	The probability that each exposed individual enters the symptomatic compartments	0.82	Ref [11]
1/*γ*_*I*_	The average duration of the infection of symptomatic infectious	12	Ref [10]
1/*γ*_*A*_	The average duration of the infection of asymptomatic infectious	7	Ref [10]
1/*γ*_*C*_	The average duration of the infection of home isolated symptomatic infectious	11	Ref [10]
1/*γ*_*Q*_	The average isolated period of quarantine isolated symptomatic infectious	14	Ref [10]
1/*γ*_*F*_	The average hospital day of symptomatic infectious in Fangcang shelter hospitals	20	Ref [10]
1/γ_H_	The average hospital day of symptomatic infectious in hospitals	10	Ref [10]
*α*_*I*_	Disease-induced death rate of symptomatic infectious estimated	0.0087 (95%CI: 0.007–0.01)	Estimated
*α*_*H*_	Disease-induced death rate of symptomatic infectious in hospitals	0.0057 (95%CI: 0.0051–0.0061)	Estimated
1/*δ*_ic_	The average day from symptom onset to clinical diagnosed after Jan 23	1	Ref [10]
1/*δ*_*cfh*_	The average day from clinical diagnosed to laboratory confirmed	1	Ref [10]
*δ*_*cq*_	The moving rate from community isolation to quarantine points isolation	0.2 (95%CI: 0.19–0.21)	Estimated
*ρ*_*Q*_	The deteriorating rate from mild or moderate to severe illness in quarantine points	0.2	Ref [12]
*ρ*_*F*_	The deteriorating rate from mild or moderate to severe illness in Fangcang shelter hospitals	0.05	Ref [12]
*θ*	The ratio of the beds used to isolate symptomatic infectious individuals in quarantine points	0.09 (95%CI: 0.08–0.11)	Estimated

Note: Reference: Ref

#### Basic reproduction number (BRN) for exposed, asymptomatic, symptomatic, and community isolated infected individuals

The basic reproduction number (BRN), defined as the expected average number of secondary cases in a completely susceptible population through a typical infective individual during the infectious period, is one of the most significant concepts in population biology [13, 14]. More importantly, it often determines the threshold behavior of many epidemic models. A disease typically dies out if the BNR is less than unity and spreads in the population otherwise. Hence, this parameter is commonly used to measure the effort required to control the spread of an infectious disease in epidemiology. We applied the next generation matrix to estimate the BRN, ${\mathcal{R}}_0(t)$ with control measures in forcing as follows:

$$ {\mathcal{R}}_0(t)=\Big\{\begin{array}{ll}\frac{{\rho \beta}_0}{\gamma_I+{\alpha}_I}+\frac{f_E{\beta}_0}{\sigma }+\frac{\left(1-\rho \right){f}_A{\beta}_0}{\gamma_A},& \mathrm{BeforeJan}23,\\ {}\frac{\rho \beta (t)}{\gamma_I+\delta +{\alpha}_I}+\frac{f_E\beta (t)}{\sigma }+\frac{\left(1-\rho \right){f}_A\beta (t)}{\gamma_A}+\frac{f_C\rho \delta \beta (t)}{\left({\gamma}_I+{\alpha}_I+\delta \right)\left({\gamma}_C+\left({C}_Q(t)+{C}_F(t)+{C}_H(t)\right)/C(t)\right)},& \mathrm{AfterJan}23.\end{array}\operatorname{} $$

In addition, the BRN of exposed, asymptomatic, symptomatic, and community- isolated infected cases are as follows:

$$ {\mathcal{R}}_0^E(t)=\left\{{\displaystyle \begin{array}{ll}\frac{f_E{\beta}_0}{\sigma },& \mathrm{Before}\ \mathrm{Jan}\ 23,\\ {}\frac{f_E\beta (t)}{\sigma },& \mathrm{After}\ \mathrm{Jan}\ 23,\end{array}}\kern0.5em {\mathcal{R}}_0^A(t)=\right\{{\displaystyle \begin{array}{ll}\frac{\left(1-\rho \right){f}_A{\beta}_0}{\gamma_A},& \mathrm{Before}\ \mathrm{Jan}\ 23,\\ {}\frac{\left(1-\rho \right){f}_A\beta (t)}{\gamma_A},& \mathrm{After}\ \mathrm{Jan}\ 23,\end{array}}{\mathcal{R}}_0^I(t)=\Big\{{\displaystyle \begin{array}{ll}\frac{\rho {\beta}_0}{\gamma_I+{\alpha}_I},& \mathrm{Before}\ \mathrm{Jan}\ 23,\\ {}\frac{\rho \beta (t)}{\gamma_I+\delta +{\alpha}_I},& \mathrm{After}\ \mathrm{Jan}\ 23,\end{array}} $$

and

$$ {\mathcal{R}}_0^C(t)=\frac{f_C\rho \delta \beta (t)}{\Big(\left({\gamma}_I+{\alpha}_I+\delta \right)\left({\gamma}_C+\left({C}_Q(t)+{C}_F(t)+{C}_H(t)\right)/C(t)\right)},\mathrm{After}\ \mathrm{Jan}\ 23. $$

The BRNs and their confidence intervals were calculated from the above formulas based on the 1000 groups of estimated values.

#### Patient and public involvement

Patients were not involved in the design of this study.

## Results

### SEIAR and SEIA-CQFH models simulated two-period epidemics

**Fig. 2:**
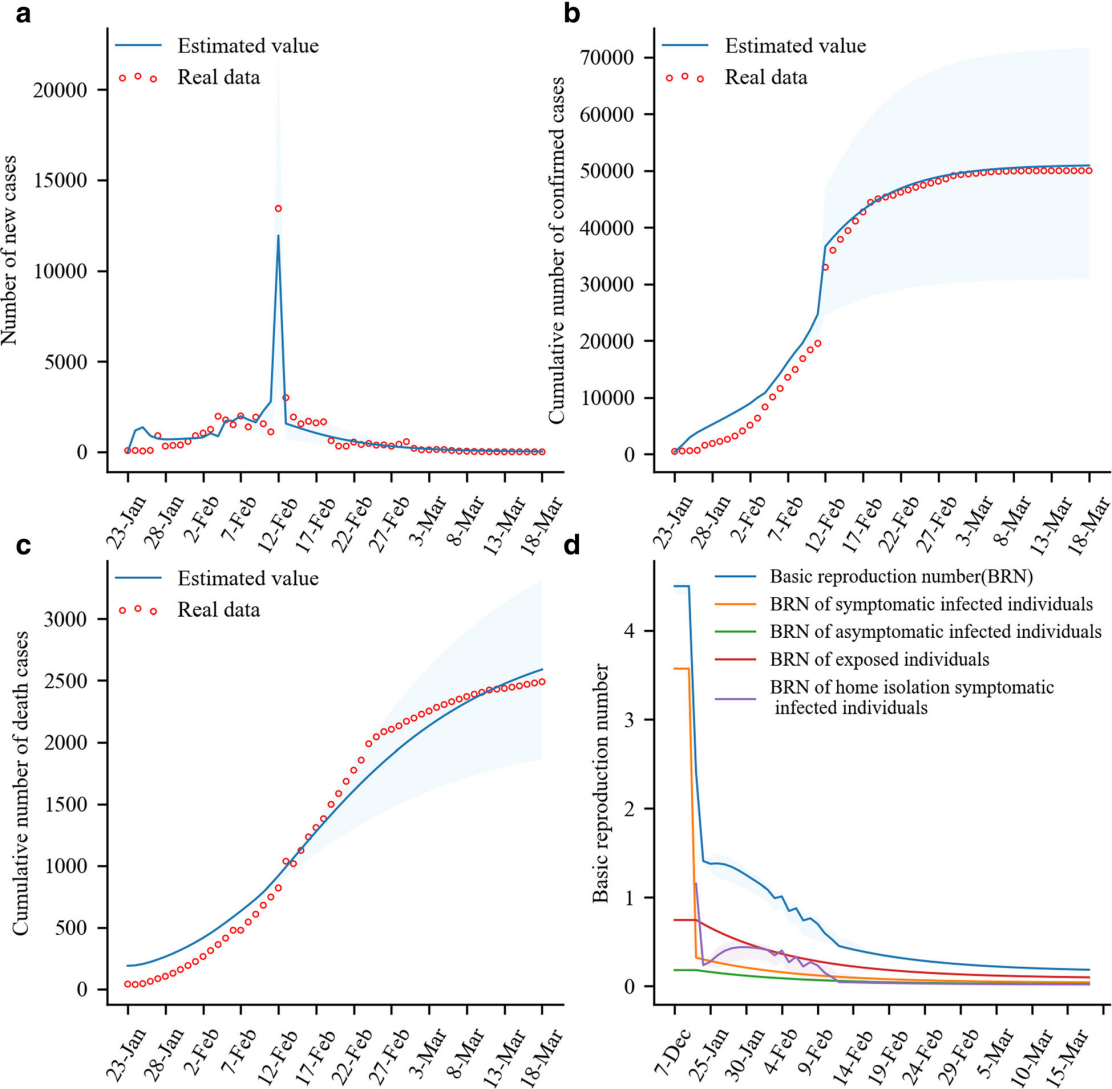
Number of COVID-19 cases and the BRN simulated by SEIAR and SEIAR-CQFH models. Panel **a**: Comparison between number of new cases simulated by model and real data. Panel **b**: Comparison between cumulative number of cases simulated by model and real data. Panel **c**: Comparison between number of deaths simulated by model and real data. Panel **d**: The BRNs of different types of cases

The numbers of newly confirmed cases, cumulative-confirmed cases, and deaths (0, 50,005, and 2496) until March 18, 2020 reported in Wuhan are basically consistent with the models simulated results (21, 50,926, and 2590), indicating that the real data and values predicted by the model were well simulated (Fig. 2 a-c). In addition, after adopting various prevention and control measures, although the BRN fluctuated slightly from February 3, 2020 to February 9, 2020, the overall trend was as follows: the BRN decreased from 4.50 on January 22, 2020 to 0.18 on March 18, 2020. Specifically, the BRNs of symptomatic infected individuals, asymptomatic infected individuals, and exposed individuals decreased from 3.57, 0.18, and 0.75 on January 23, 2020 to 0.04, 0.02, and 0.10 on March 18, 2020. In addition, although the BRN of community-isolation symptomatic infected individuals increased slightly from 0.24 on January 24, 2020 to 0.41 on February 2, 2020, the BRN continued to decrease from 0.27 on February 8, 2020 to 0.02 on March 18, 2020 (Fig. 2d). In addition, the SEIAR-CQFH model simulated the transmission rule of the epidemic after January 23, 2020, and found that the increase in the numbers of cumulative confirmed cases and deaths was smooth, except for February 12, 2020 (Fig. 2a). The reason for the surge was that on February 12, 2020, clinical diagnosis cases were enrolled as confirmed cases.

### Assessment of Fangcang shelter hospitals

**Fig. 3:**
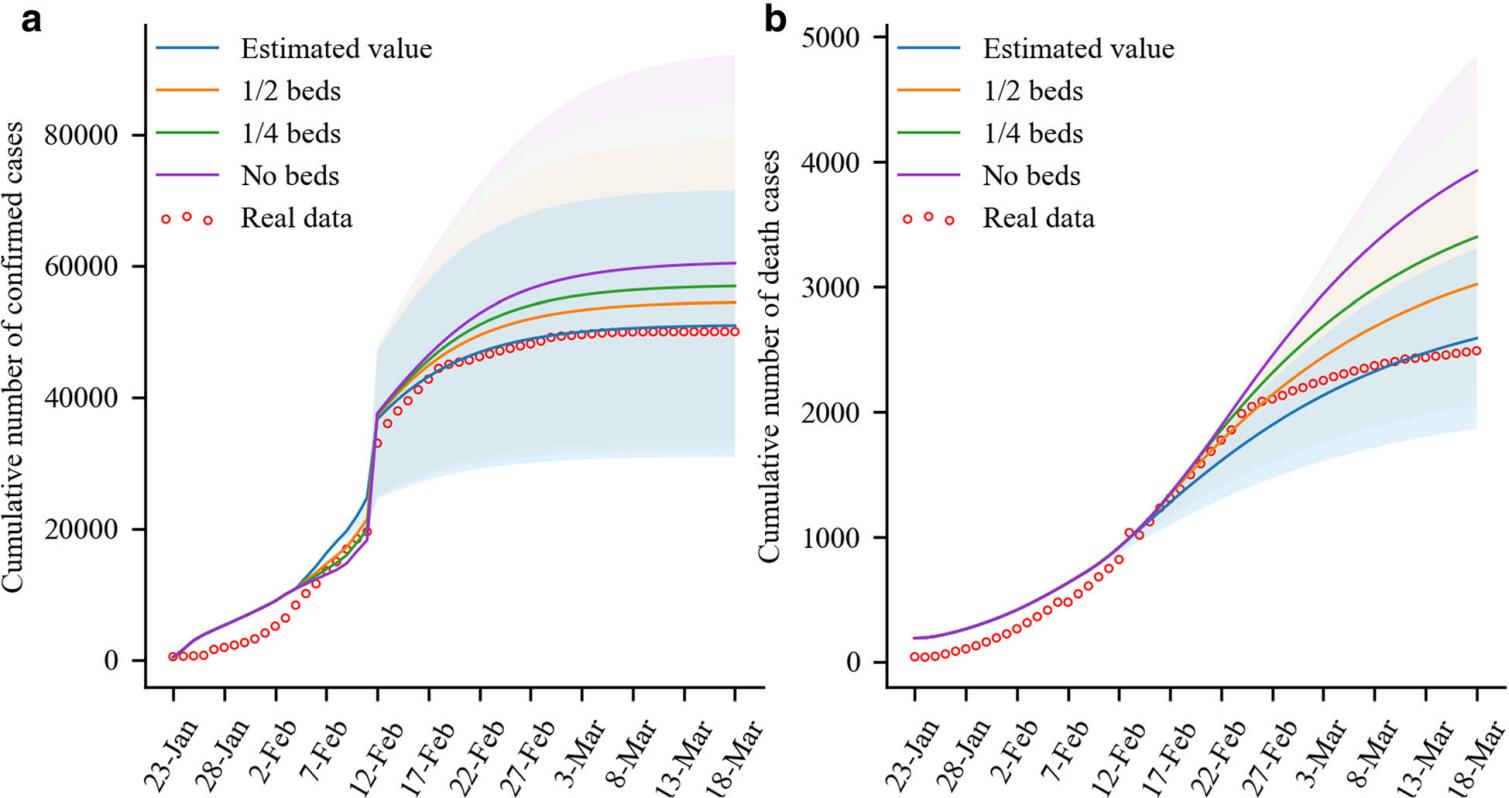
Cumulative numbers of cases and deaths vary with the beds number in Fangcang shelter hospitals. Panel **a**: Cumulative number of COVID-19 cases. Panel **b**: Cumulative number of COVID-19 deaths

The SEIAR-CQFH model simulated the number of beds in Fangcang shelter hospitals. With this model, we confirmed that if the number of beds was reduced by 1/2 or 3/4, the growth ranges of the numbers of cumulative confirmed cases and deaths increased obviously, especially the cumulative deaths. Specifically, if the numbers of beds in Fangcang shelter hospitals were 0, 1/4, 1/2, and normal, the cumulative number of cases on March 18, 2020 would be 60,389, 56,924, 54,430, and 50,925 (real data: 50,005), respectively. By March 18, 2020, there would be 18.58, 11.78, and 6.88% more cumulative confirmed cases in the cases of 0, 1/4, and 1/2 beds, compared to the condition with normal beds (Fig. 3a). Compared to the cumulative number (425) of cases on January 23, 2020, the month-on-month growths in the cases of 0, 1/4, 1/2, and normal beds were 12,533.73, 12,082.60, 11,683.11, and 11,049.31%, respectively (Fig. 3a). In addition, if the numbers of beds in Fangcang shelter hospitals were 0, 1/4, 1/2, and normal, the numbers of deaths on 18 March, 2020 were 3929, 3399, 3019, and 2590 (real data: 2495), respectively. By March 18, 2020, there would be 51.73, 31.25, and 16.59% more cumulative deaths in the cases of 0, 1/4, and 1/2 beds, respectively, compared to the condition with normal beds. Compared to the death number (190) on January 23, 2020, the month-on-month growths in the cases of 0, 1/4, 1/2, and normal beds were 954.56, 927.92, 872.48, and 779.35%, respectively (Fig. 3b ).

### Assessment of designated hospitals

**Fig. 4:**
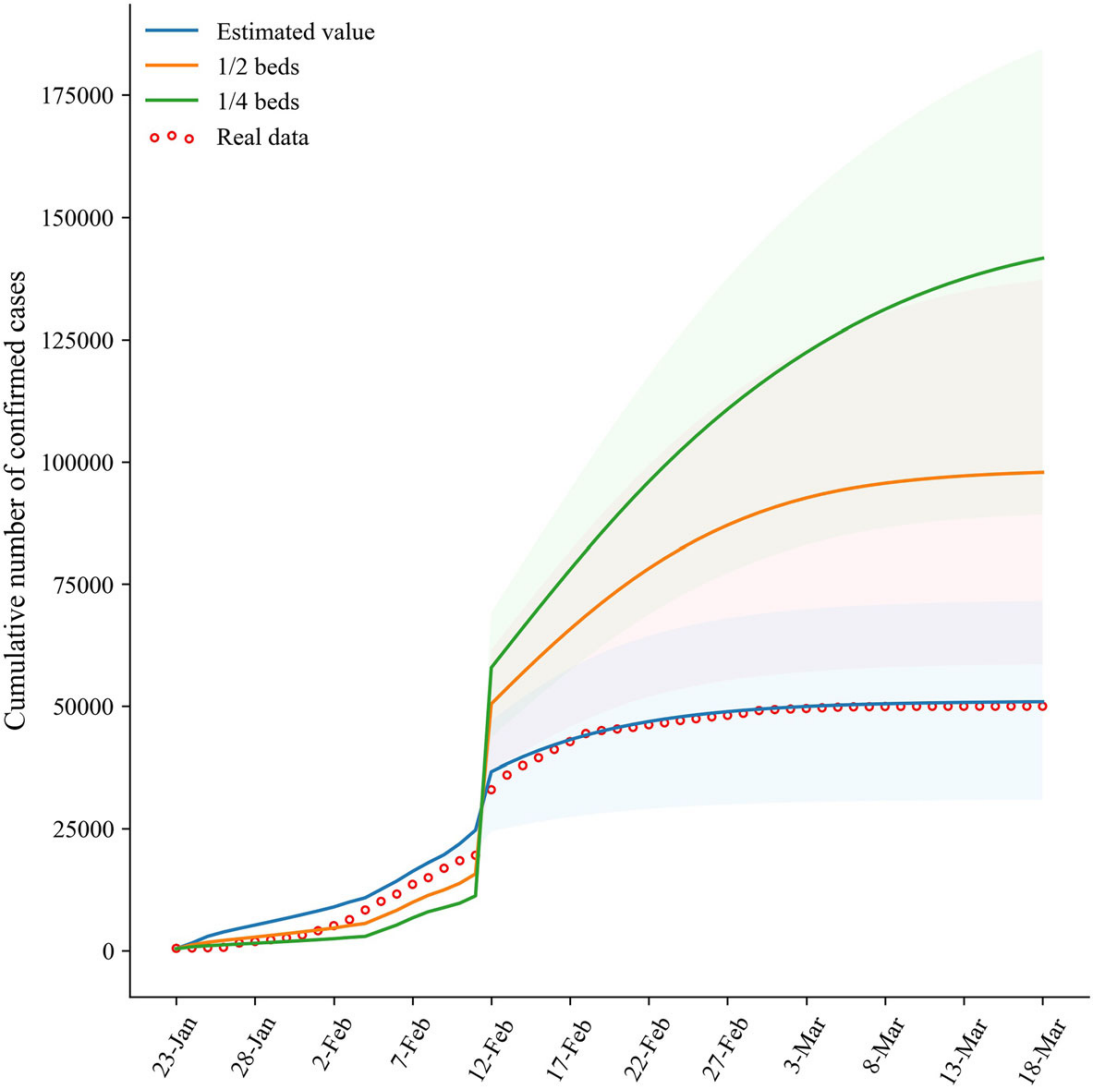
Cumulative number of COVID-19 cases vary with the number of beds in designated hospitals

The impact of the number of beds in designated hospitals is similar to that in Fangcang shelter hospitals in the COVID-19 epidemic; however, it is important to note that reducing the number of beds in designated hospitals would lead to a more significant increase in confirmed cases. Specifically, if the numbers of beds in designated hospitals are 1/4, 1/2, and normal, the cumulative numbers of confirmed cases on March 18, 2020 would be 141,594, 97,829, and 50,926 (real data: 50,005), respectively. By March 18, 2020, there would be 178.04 and 92.1% more cumulative confirmed cases for 1/4 and 1/2 beds, respectively, compared to the condition with normal beds. Compared to the cumulative number of cases (425) on January 23, 2020, the month-on-month growths for 1/4, 1/2, and normal beds were 23,234.81, 18,784.59, and 11,049.31%, respectively. Although the number of cases increased slightly with the increase in bed size in the early period, the increase in the number of beds significantly inhibited the increase in the number of cases in the later period (Fig. 4).

### Assessment of the joint measures led by Fangcang shelter hospitals

**Fig. 5:**
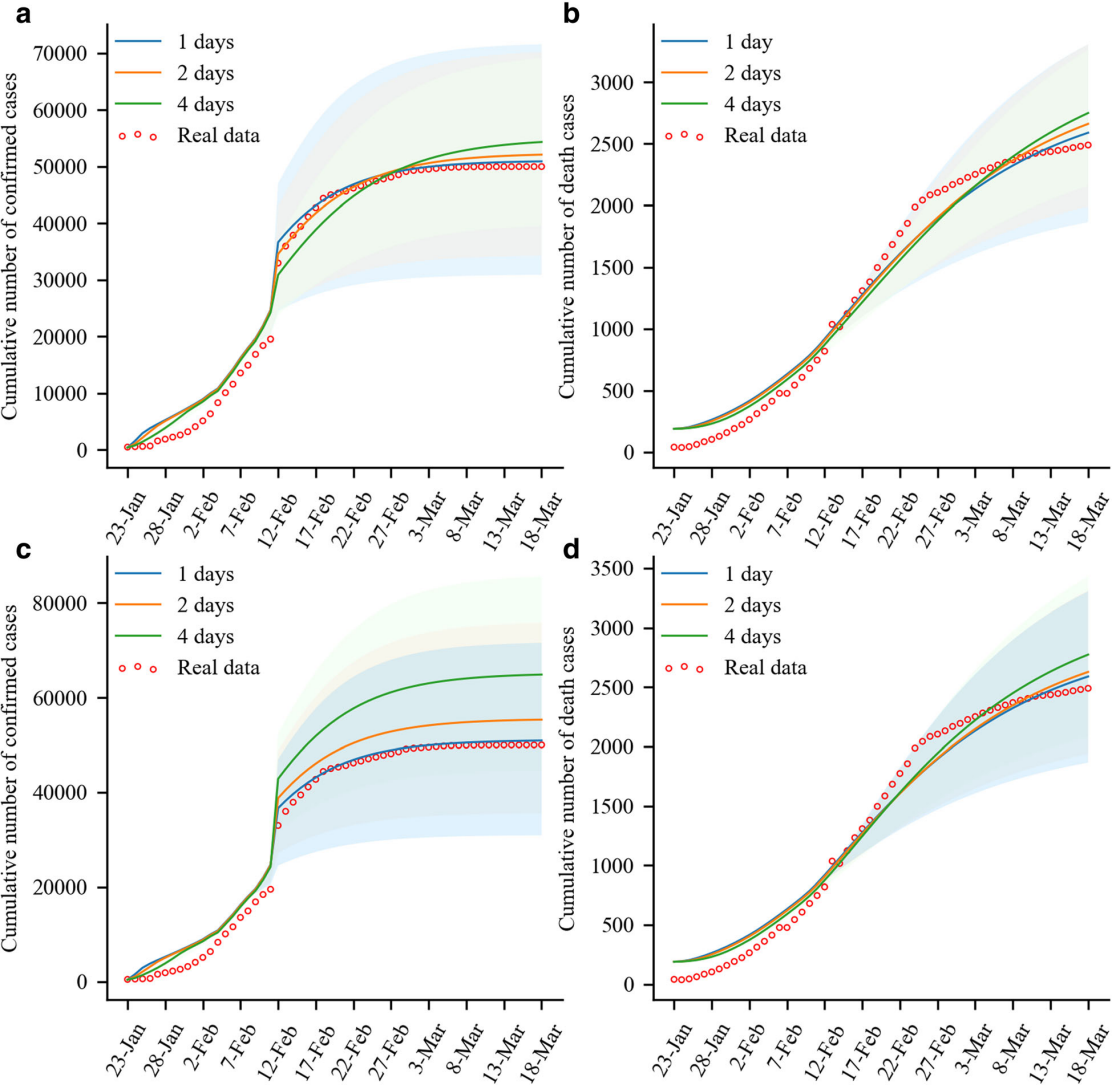
Cumulative numbers of COVID-19 cases and deaths vary with the time intervals. Panel **a**: Cumulative number of cases varies with the time intervals from illness onset to hospital visit. Panel **b**: Cumulative number of deaths varies with the time intervals from illness onset to hospital visit. Panel **c**: Cumulative number of cases varies with the time intervals from hospital visit to diagnosis. Panel **d**: Cumulative number of deaths varies with the time intervals from hospital visit to diagnosis

We used the SEIAR-CQFH model to estimate the impact of the time interval from illness onset to hospital visit at 1 day, 2 days, and 4 days in the COVID-19 epidemic and found that the time interval from illness onset to hospital visit was of importance for the epidemic. Compared to 1 and 2 days, the time interval from illness onset to hospital visit was 4 days, resulting in a significant increase in the number of confirmed cases and deaths. Specifically, the cumulative numbers of cases and deaths as of March 18, 2020 were 54,350 and 2750, respectively, an increase of 6.73, 4.29 and 6.19%, 3.31% in comparison with that of 1 day and 2 days, respectively (Fig. 5a-b).

The increased impact of the time interval from hospital visit to diagnosis was more pronounced on the numbers of confirmed cases and deaths. Specifically, before February 12, 2020, the time interval from hospital visit to diagnosis had lesser effect on the cumulative numbers of cases and deaths. After February 12, 2020, the shorter the time interval from hospital visit to diagnosis, the fewer the accumulated cases. As of March 18, 2020, the cumulative numbers of cases were 50,926, 55,334, and 64,863, respectively, for 1 day, 2 days, and 4 days, respectively. In addition, the cases were more significantly affected after February 22, 2020, that is, the shorter the time interval from hospital visit to diagnosis, the fewer the number of deaths, and the gap increased with time. As of March 18, 2020, the total numbers of deaths were 2590 (real data: 2496), 26,280 and 27,740 for 1, 2, 4 days, respectively (Fig. 5c-d).

**Fig. 6:**
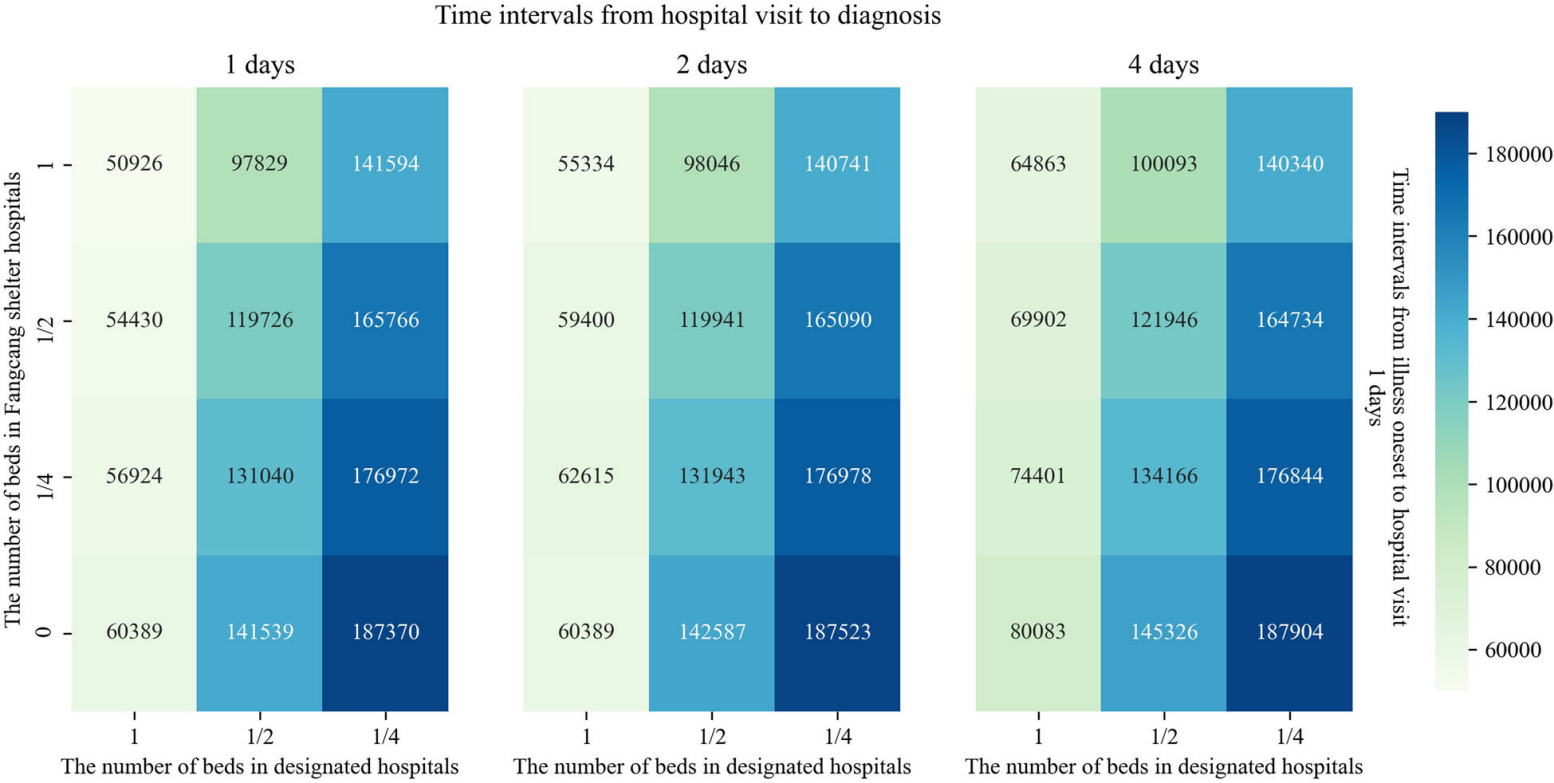
Cumulative number of COVID-19 cases varies with the joint prevention and control measures

In general, in view of the severity and public concern of the COVID-19 epidemic, the onset individuals usually visit the hospitals soon; other medical services including the time for diagnosis, number of beds in Fangcang shelter hospitals, and number of beds in designated hospitals were evaluated and required by the Chinese government. Therefore, we put the dynamic change of the above variables, except the variables for illness onset to visit hospitals, into the model to simulate the effect of changes of multiple variables on the cumulative number of cases to evaluate the joint measures. The results indicate that the numbers of beds in Fangcang hospitals and designated hospitals are normal, and both time intervals from illness onset to hospital visit and diagnosis are 1 day; the cumulative number of cases is the fewest and basically consistent with the real data (50,926; real data: 50,005). The number of Fangcang shelter hospitals is 0, the number of beds in designated hospitals is 1/4, the time interval from onset to hospital visit is 1 day, and the time interval from hospital visit to diagnosis is 4 days, the cumulative number of cases would be the highest (187,904). For the other combinations’ types of medical services, the cumulative number of cases caused is higher than the actual number of prevention and control measures (Fig. 6).

## Discussion

Considering the asymptomatic cases in the classic transmission dynamic model SEIR, we simulated the transmission rule of epidemic using an extended SEIAR model before and after January 23, 2020 and added four cabins, including community isolation, quarantine point isolation, Fangcang shelter hospitals, and designated hospitals to construct the SEIAR-CQFH model. The simulated results indicate that the numbers of new cases (21), cumulative confirmed cases (50,926), and deaths (2590) until March 18, 2020 were estimated by two-stage SEIR and SEIAR-CQFH models, which are close to the data published by the government (0, 50,005 and 2496) [1, 3, 5]. In addition, for any day from December 7, 2019 to March 18, 2020, the real data was matched with the simulated data, indicateding that the two-stage models were appropriate for COVID-19 transmission. In addition, the BRN decreased from 4.50 on January 23, 2020, to 0.18 on March 18, 2020, which is also consistent with other reports [15–17]. In addition, the BRNs of symptomatic infected cases, asymptomatic infected cases, exposed individuals, and isolated infected cases decreased from January 23, 2020 to March 18, 2020. The time when the BRN began to decline was basically the same as the time when the national medical teams assisted Wuhan and the lockdown of Wuhan [18, 19] and indicates that the effectiveness of joint prevention and control strategies adopted by the Chinese government. Therefore, we used the SEIAR-CQFH model to quantitatively assess the measures of Fangcang shelter hospitals, designated hospitals, and the time intervals from onset illness to diagnosis in the COVID-19 epidemic after January 23, 2020 in Wuhan city in mainland China.

In the joint prevention and control of the COVID-19 epidemic, Fangcang shelter hospitals play important isolation and triage roles as intermediate platforms. At the beginning of February 2020, designated hospitals in Wuhan did not have enough beds for COVID-19 patients, especially for thousands of patients with mild to moderate COVID-19 symptoms [2, 3]. Mild and moderate patients could be isolated in the community; however, epidemiological studies revealed that in China, COVID − 19 has a high rate of intrafamily transmission [2, 10, 20–23], and more than 50% of COVID-19 patients had at least one family member with the disease [2]. In addition, it is difficult to monitor disease progress in community isolation, and asymptomatic infected individuals may deteriorate to having mild and moderate symptoms [21–23]. Our study also confirmed that the BRN for community isolation symptomatic infected individuals increased from January 24 to February 2, 2020, while other BRNs decreased. This indicates that community isolation was not effective. On Feb 2, 2020, Wuhan asked community isolation individuals, newly suspected individuals, and close contacts to move to designated point isolations. After 3 days, on February 5, 2020, Wuhan successively opened 16 Fangcang shelter hospitals to treat mild and moderate patients. This implementation of this measure was conducive to the rapid isolation and triage of mild and moderate cases. Therefore, from February 5, 2020, the BRN of community-isolated symptomatic infected individuals exhibited a continuous downward trend.

In addition to isolation, triage, basic medical care, frequent monitoring, and rapid referral were also the original intentions of the establishment of the Fangcang shelter hospitals [2, 3]. Our study also confirmed the important role of Fangcang shelter hospitals in the early treatment of COVID-19, and the results show that without Fancang shelter hospitals, the cumulative numbers of cases and deaths would increase by 18.58 and 51.73% by March 18, 2020. In addition, if the number of beds was reduced to 1/2 or 1/4, the cumulative numbers of cases and deaths would increase by 6.88 and 11.78% or 16.59 and 31.25%, respectively. Moreover, one of the important functions of Fancang shelter hospitals was monitoring and rapid referral [2], which enabled severe COVID-19 cases to be treated in the shortest time and increased the survival possibility. The treatment of severe cases was inseparable from the designated hospitals, designated hospitals also played an important role as a high-level platform in hierarchical prevention and control. Our study also showed that if the number of beds in the designated hospitals decreased by 1/2 or 1/4, the number of COVID-19 cases would increase significantly from 50,926 to 97,829 and 141,594, respectively. After January 25, 2020, with continuous increase in medical materials and medical staff, the number of beds in designated hospitals increased, which increased the treatment opportunities of severe cases and reduced the death of severe cases of COVID-19.

Shortening the time interval between hospital transfers can increase the survival possibility in severe cases, similarly, shortening the time intervals from illness onset to hospital visit and confirmation can also reduce the deaths and transmission. Our study showed that the numbers of deaths and culminative cases significantly decreased after reducing the time intervals from illness onset to hospital visit and from hospital visit to confirmation. We used models to simulate the effect of joint measures and found that if the number of beds in Fangcang hospitals and designated hospitals were normal, and both the time intervals from illness onset to hospital visit and diagnosis were 1 day, the cumulative number of cases was the fewest and basically consistent with the real data (50,926; real data: 50,005). Therefore, the measure at that period was optimal; if Fangcang shelter hospitals were not established, the number of beds reduced 1/4 and the time interval was 4 days, the cumulative number of cases would increase by 268.97%. This further verifies the importance of joint measures in COVID-19 epidemic prevention and control, and thus, the measures deserve to be rolled out globally. In addition, the numbers of beds in Fangcang shelter hospitals and designated hospitals and the time interval of diagnosis were the best combination, as determined by the detailed and professional evaluation by the Chinese government, it is the best and fastest choice after the full evaluation of the materials, personnel, and other conditions under the increasingly severe situation of the COVID-19 epidemic. Our study also verified this result. However, we must consider and sum up how we can learn from the COVID-19 epidemic response and improve the ability to deal with emerging infectious diseases, especially when medical resources are limited.

This study has some limitations. First, it did not quantitatively assess the effectiveness of community isolation and quarantine point isolation because of the difficulty encountered in collecting the related data set. Second, we were unable to collect the real-time number of beds in the central isolation point, and we used a fixed number published by the National Health and Planning Commission.

In conclusion, our study provides a detailed quantitative assessment of the effects of Fangcang shelter hospitals, designated hospitals, and time intervals from illness onset to hospital visit and diagnosis of COVID-19 in Wuhan city, mainland China, especially the role of Fangcang shelter hospitals. The results indicate that Fangcang shelter hospitals, similar to designated hospitals, played an irreplaceable contribution to the control of the COVID-19 epidemic; moreover, the combination of measures, including the normal number of beds in Fangcang hospitals, was optimum, making the prevention and control strategies more effective. Lastly, although the COVID-19 epidemic has been basically brought under control in China and Fangcang shelter hospitals have been closed, we still cannot take it lightly. We should summarize the prevention and control experience of COVID-19, and provide more scientific methods for the Chinese and even global people in response to the outbreak of emerging infectious diseases, especially for countries and regions with limited medical resources.

## Supplementary Information

**Additional file 1: Figure S1.** The number of maximum open beds of Fangcang shelter hospitals (A), designated hospitals (B) and the number of physicians (C) supporting Hubei from outside Hubei. **Figure S2.** The maximum open beds per day of quarantine points, Fangcang shelter hospitals and designated hospitals.

## Data Availability

Our data are from publicly published data, have no privacy implications and can be founded in http://wjw.wuhan.gov.cn/.
