# Supplementary material for: Quantitative assessment of the effectiveness of joint measures led by Fangcang shelter hospitals in response to COVID-19 epidemic in Wuhan, China
Source: BMC Infect Dis. 2021 Jul 1;21:626. doi: 10.1186/s12879-021-06165-w (PMC8245925; doi:10.1186/s12879-021-06165-w)
Supplement: Supplementary file 1 — Additional file 1: Figure S1. The number of maximum open beds of Fangcang shelter hospitals (A), designated hospitals (B) and the number of physicians (C) supporting Hubei from outside Hubei. Figure S2. The maximum open beds per day of quarantine points, Fangcang shelter hospitals and designated hospitals. [file 12879_2021_6165_MOESM1_ESM.docx]

**Supplementary Text: “**Quantitative assessment of the effectiveness of joint measures led by the Fangcang shelter hospitals in response to COVID-19 epidemic in Wuhan, China

Hui Jiang*1,2, Pengfei Song*3, Siyi Wang*4, Shuangshuang Yin3, Jinfeng Yin1, Chendi Zhu1,2, Chao Cai5, Wangli Xu†4, Weimin Li†1,2,6

1. Beijing Chest Hospital, Capital Medical University, Beijing, 101149, China.

2. Beijing Tuberculosis and Thoracic Tumor Research Institute, Beijing, 101149, China.

3. School of Mathematics and Statistics, Xi'an Jiaotong University, Xi'an, ShaanXi, 710049, China.

4. Center for Applied Statistics, School of Statistics, Renmin University of China, Beijing, 100872, China.

5. Beijing Youan Hospital, Capital Medical University, Beijing, 100069, China.

6. Beijing Municipal Key Laboratory of Clinical Epidemiology, School of Public Health, Capital

Medical University, Beijing, 100069, China.

Co-corresponding author: Prof. Weimin Li, Prof. Wangli Xu

Email: lwm_18@aliyun.com, xwlbnu@163.com

Tel & Fax: 86-10-8950-9359

* Contributed equally to this work.

† Co-senior authors on this study.

**1 Data Sources**

The newly confirmed, cumulative confirmed and cumulative death cases of COVID-19 in Wuhan from Jan 23 to
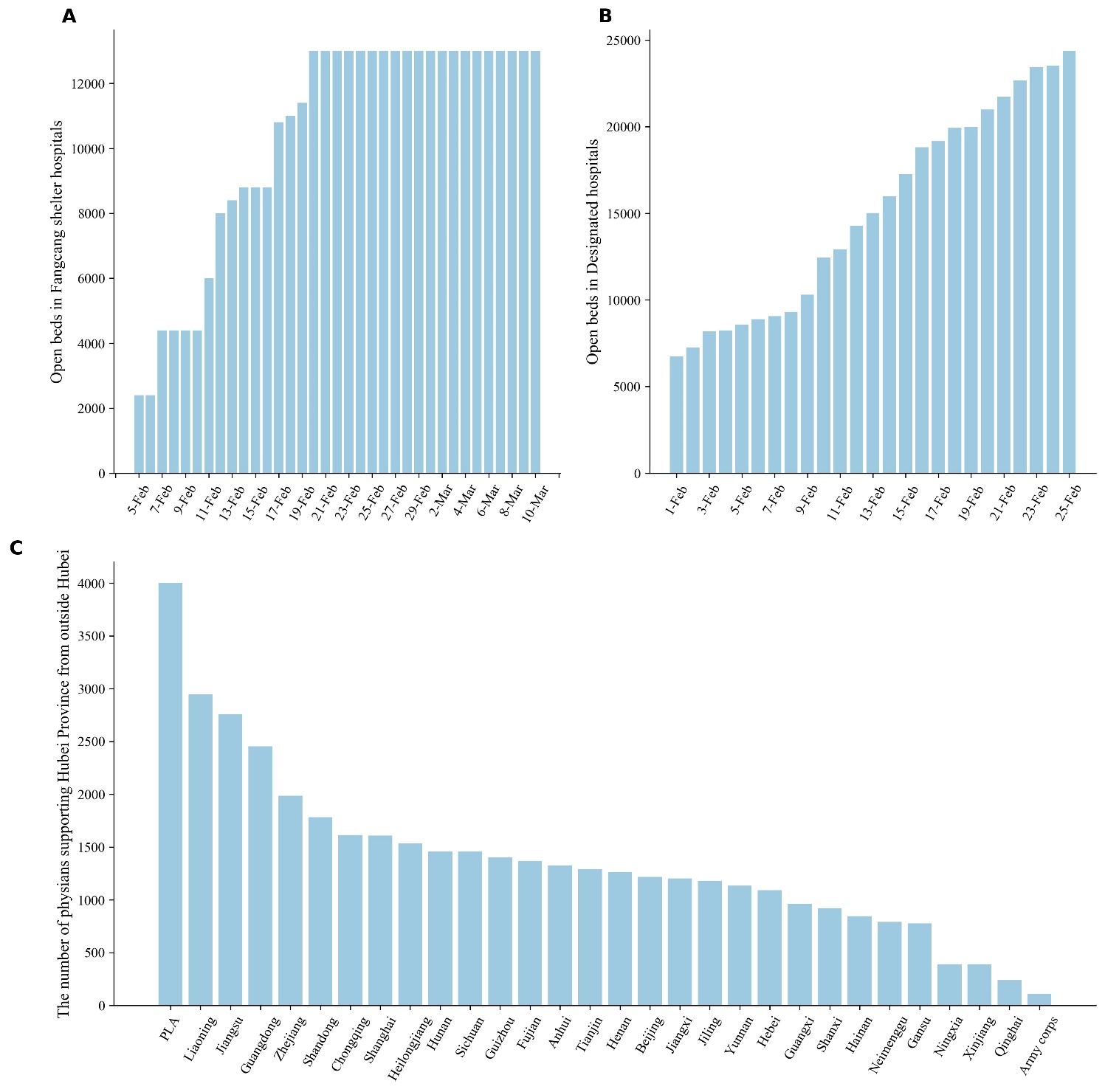
 **Fig 1. The number of maximum open beds of Fangcang shelter hospitals (A), designated hospitals (B) and the number of physicians (C) supporting Hubei from outside Hubei.**

March 18 were collected from the National Health Commission of the People’s Republic of China ^1^ and World Health Organization ^2^. The number of the maximum open beds of Fangcang shelter hospitals from Feb 5 to

March 10 were collected from [4] and Wuhan Municipal Health Commission ^3^, showed in Figure 1A. The number of the maximum open beds in designated hospitals on Jan 23 and from Feb 1 to Feb 25 were obtained from Wuhan Municipal Health Commission ^3^, and the data from Feb 1 to Feb 25 was showed in Figure 1B, and the data in Jan 23 equal to 2613. The number of the maximum open beds of quarantine points on Feb 5 (12571) is obtained from Wuhan Municipal Health Commission ^3^. The number of physicians supporting Hubei province from outside Hubei was obtained from [5], showed in Figure 1C.

# 2 SEIAR and SEIAR-CQFH warehouse models

The starting date of the epidemic is fixed on Dec 7 ^6^. We use the date of Wuhan locked down to divide the Wuhan epidemic into two time periods, i.e., Dec 7 to Jan 22, and Jan 23 to March 18, and use SEIAR and SEIA-CQFH warehouse models to simulate the two-stage Wuhan epidemic, respectively. The warehouse model has been widely applied in mathematical epidemiology for a long time since it was forwarded by Kermack and McKendrick in 1927 ^7^. It includes several basic and improved models such as SIR, SIS, SEIR and so on ^8^. For the first stage epidemic from Dec 7 to Jan 22, in order to include the asymptomatic infected individuals, we extend the basic SEIR (Suspected-Exposed-Infected-Recovered) model to SEIAR model as follows:



$$\left\{\begin{matrix} \frac{\text{d}S}{\text{dt}}=-\beta_{0}(I+f_{A}A+f_{E}E)\frac{S}{N}, \\ \frac{\text{d}E}{\text{dt}}=\beta_{0}(I+f_{A}A+f_{E}E)\frac{S}{N}-\sigma E, \\ \frac{\text{d}I}{\text{dt}}=\rho\sigma E-\gamma_{I}I-\alpha_{I}I, \\ \frac{\text{d}A}{\text{dt}}=(1-\rho)\sigma E-\gamma_{A}A, \\ \frac{\text{d}R}{\text{dt}}=\gamma_{I}I+\gamma_{A}A, \end{matrix}\right.$$

where $S(t),E(t),I(t),A(t),R(t)$and$N=S(t)+E(t)+I(t)+A(t)+R(t)$ are the number of susceptible (S), exposed (E), symptomatic infectious (I), asymptomatic infectious (A), recovered individuals (R) and total population in Wuhan at time $t$, respectively. The functions $S(t),E(t),I(t),A(t),R(t)$ dependent on $t$ are simply denoted as $S,E,I,A,R$ in SEIAR model. Here, the susceptible individuals are infected by exposed, asymptomatic infectious and symptomatic infectious individuals with rates $f_{E}\beta_{0},f_{A}\beta_{0}$ and $\beta_{0}$ respectively, and become exposed. Each exposed individual, entering the symptomatic compartments with the rate $\rho\sigma$or becoming asymptomatic with the rate $(1-\rho)\sigma$, becomes infectious. The infected individuals are recovered with a rate $\gamma_{I}$. The parameter $\alpha_{I}$ is the death rate of symptomatic infectious disease. The first stage SEIAR model simulation was based on the following assumptions A1-A6:

**A1**. The whole population in Wuhan is susceptible ^9^;

**A2**. The disease induced death rate of asymptomatic infectious individuals is zero;

**A3**. The susceptible and the infectious are homogenous in the population and population migration is not included;

**A4**. The epidemic is originated from one single case;

**A5**. The intervention effect was not included in the first stage of COVID-19 epidemic in Wuhan.

**A6**. The susceptible individuals can be infected by exposed and asymptomatic infectious ^10^.

The aforementioned assumptions are in line with the reality in Wuhan, China.

For the second stage epidemic from Jan 23 to March 18, we extend SEIAR model to include clinically diagnosed symptomatic individuals in community isolation (C), quarantine points isolation (Q), Fangcang shelter hospitals (F) and designated hospitals (H). The SEIAR-CQFH model is described as follows:

where $S(t),E(t),I(t),A(t),R(t)$and$N$ have the same meanings as that in SEIAR model. $C(t),Q(t),F(t)$ and $H(t)$ are the number of clinically diagnosed symptomatic individuals in community isolation, quarantine point isolation, Fangcang shelter hospitals and designated hospitals at time $t$. Similarly, as SEIAR model, and we suppress all the functions dependent on $t$ in SEIAR-CQFH model for notations simply.

For SEIAR-CQFH model, symptomatic infectious individuals is clinically diagnosed and enters community isolation class with a rate $\delta$. Home isolated symptomatic infectious individuals will be moved to Fangcang shelter hospitals or designated hospitals if beds are available, and the number of people moved from community isolation to Fangcang shelter hospitals or designated hospitals per day are denoted as $C_{F}(t),C_{H}(t)$, respectively. If beds in Fangcang shelter hospitals or designated hospitals are not enough, community isolation individuals will be moved to quarantine points with the number of people per day denoted as $C_{Q}(t),$ or isolated at home and recovered at rate $\gamma_{C}$. Quarantine points isolated symptomatic infectious individuals will be moved to Fangcang shelter hospitals and designated hospitals if beds are available, with the number of people moved from quarantine points to Fangcang shelter hospitals or designated hospitals per day denoted as $Q_{F}(t),Q_{H}(t)$, respectively. Otherwise, the infectious individuals in quarantine points will continue to be isolated at quarantine points and recovered at rate $(1-\rho_{Q})\gamma_{Q}$ , where $\rho_{Q}$ is the probability deteriorating from mild or moderate to severe illness and $1/\gamma_{Q}$ is the average isolated period of quarantine isolated symptomatic infectious. If symptomatic infectious individuals in Fangcang shelter hospitals deteriorate from mild or moderate to severe illness, they will be moved to designated hospitals if beds are available，and the number moved from Fangcang shelter hospitals to designated hospitals per day is denoted as $F_{H}(t)$. The second stage SEIAR-CQFH model was based on the following hypotheses:

**B1.** In quarantine points isolation and Fangcang shelter hospitals, only individuals deteriorating from mild or moderate to severe illness can be moved to designated hospitals;

**B2.** The disease induced death rate of clinically diagnosed symptomatic individuals in community isolation, quarantine points isolation, Fangcang shelter hospitals are assumed to be zero;

**B3. S**ymptomatic individuals in Fangcang shelter hospitals share the highest priority moving to designated hospitals, then quarantine points and then community isolation. Symptomatic individuals in quarantine points share the higher priority moving to Fangcang shelter hospitals than community isolation;

**B4.** The intervention began to take effect on Jan 23.

**B5.** After the interventions began to take effect, the transmission rate decreased exponentially.

**B6.** The transmission rate of symptomatic individuals and home isolated clinically diagnosed symptomatic individuals are the same.

**B7.** Only part of the beds was used to isolated real symptomatic infectious individuals in quarantine points, and we denote the rate as $\theta$.

For assumption B2, it is reasonable because clinically diagnosed symptomatic individuals deteriorating from mild or moderate to severe illness are moved to designated hospitals. Assumption B3 is rationality by considering the fact that symptomatic individuals in Fangcang shelter hospitals can receive better medical care than in quarantine points, and quarantine points better than community isolation. Assumption B7 is guaranteed since quarantine points also isolated suspected patients and close contacts ^11^. The others assumptions for SEIAR-CQFH model are mild and match with the real situations.

Based on the above-mentioned hypothesis B1-B7, we assume the baseline transmission rate after Jan 23 satisfying

$$\beta(t)=(\beta_{0}-\beta_{end})\exp(-rt)+\beta_{end}$$

and

$$\begin{matrix} F_{H}(t)=\min\{\max\{H_{max}(t)-H(t),0\},\rho_{F}\gamma_{F}F(t)\}, \\ Q_{H}(t)=\min\{\max\{H_{max}(t)-H(t)-F_{H}(t),0\},\rho_{Q}\gamma_{Q}Q(t)\}, \\ C_{H}(t)=\min\{\max\{H_{max}(t)-H(t)-F_{H}(t)-Q_{H}(t),0\},\delta_{cfh}C(t)\}, \\ Q_{F}(t)=\min\{\max\{F_{max}(t)-F(t),0\},\delta_{cfh}Q(t)\} \\ C_{F}(t)=\min\{\max\{F_{max}(t)-F(t)-Q_{F}(t),0\},\delta_{cfh}C\}, \\ C_{Q}(t)=\min\{\max\{\theta Q_{max}(t)-Q(t),0\},\delta_{cq}Q(t)\}. \end{matrix}\text{ }$$

where $H_{max}(t)$ , $F_{max}(t)$ and $Q_{max}(t)$ is the maximum open beds in designated hospitals, Fangcang shelter hospitals and quarantine points. Only if designated hospitals had vacant beds with the number mathematically denoted as $\max\{H_{max}(t)-H(t),0\}$, those symptomatic infectious individuals in Fangcang shelter hospitals deteriorating from mild or moderate to severe illness (mathematically denoted as $\rho_{F}\gamma_{F}F(t)$), would be allowed to move to designated hospitals. Hence the number per day moved from Fangcang shelter hospitals to designated hospitals was $\min\{\max\{H_{max}(t)-H(t),0\},\rho_{F}\gamma_{F}F(t)\}$. For $Q_{H}(t)$ being the number of people moved from quarantine points to designated hospitals per day, the number of vacant beds in designated hospitals at time $t$ becomes $\max\{H_{max}(t)-H(t)-F_{H}(t),0\}$, thus the number of symptomatic infectious individuals in quarantine points deteriorating from mild or moderate to severe illness ( mathematically denoted as $\rho_{F}\gamma_{F}F(t)$) moving to designated hospitals is$\min\{\max\{H_{max}(t)-H(t)-F_{H}(t),0\},\rho_{Q}\gamma_{Q}Q(t)\}$. The functions $C_{H}(t),Q_{F}(t),C_{F}(t),C_{Q}(t)$ can be similarly defined.

The first three fangcang shelter hospitals started to work on Feb 5 and provided 4000 beds, which means that $F_{max}(t)$ is zero before Feb 5th in the second stage SEIAR-CFQH model. Over the following 3 weeks, Wuhan opened an additional 13 Fangcang shelter hospitals, providing 12600 beds. The progression of bed capacity of the Fangcang shelter hospitals over time ($F_{max}(t)$ ) is showed in Fig 6.

The collected data for the maximum open beds in designated hospitals $H_{max}(t)$ is on Jan 23 and from Feb 1 to Feb 25, and that for quarantine points $Q_{max}(t)$ is on Feb 5 (12571). Hence the complete observed data is not available from Jan 23 to March 18. To determine the value of $H_{max}(t)$from Jan 23 to Feb 1, we assumed that the number of the maximum open beds in designated hospitals increased linearly from Jan 23 (2613) to Feb 1(6754). Moreover, we assume that the number of the maximum open beds per day keep the same after Feb 25 (24378). The number of the maximum open beds of quarantine points was assumed as a constant (12571) from Jan 23 to March 18. The maximum open beds in designated hospitals $H_{max}(t)$, Fangcang shelter hospitals and quarantine points $Q_{max}(t)$ over time are show in Fig. 2.


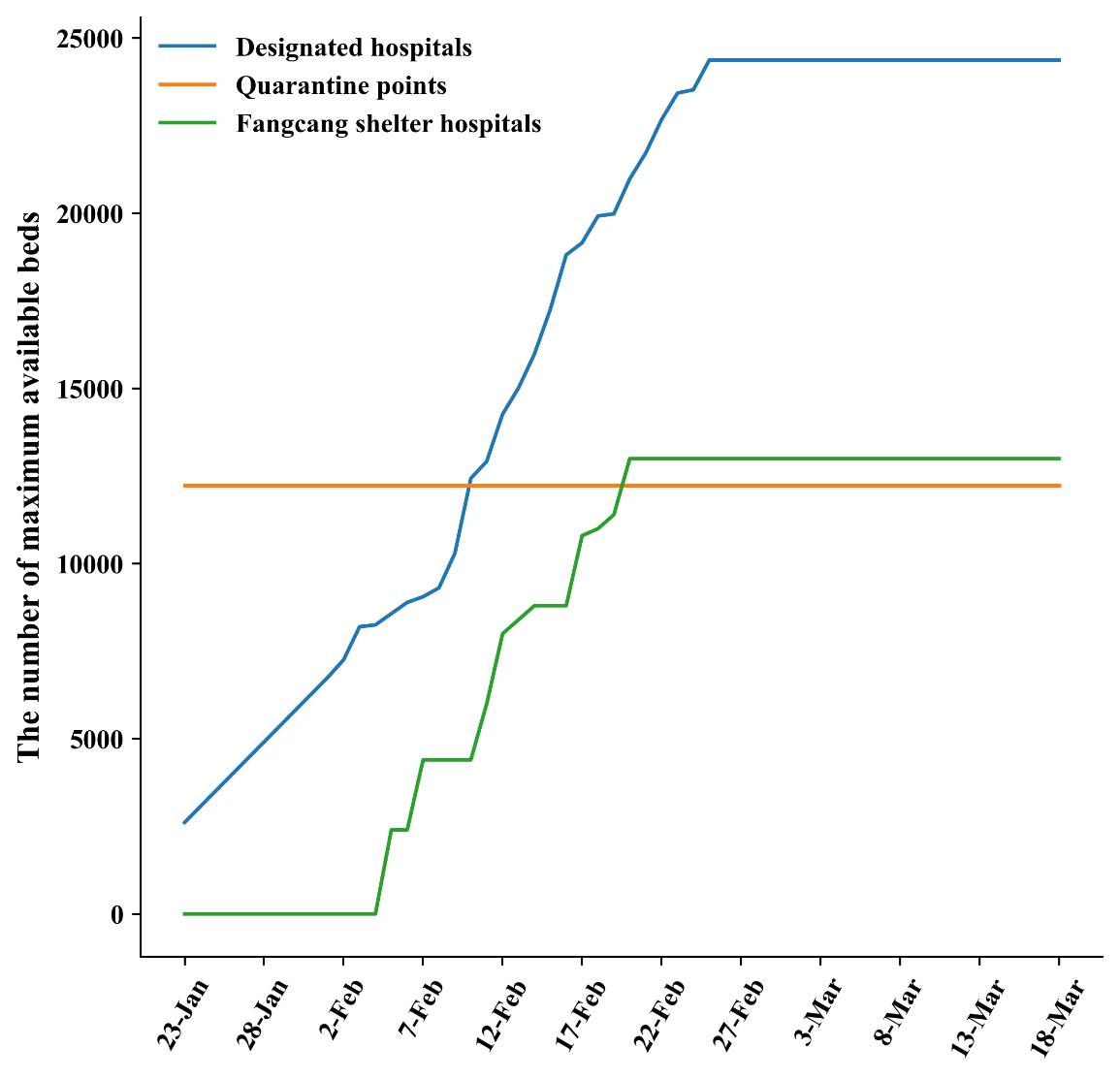


**Fig 2. The maximum open beds per day of quarantine points, Fangcang shelter hospitals and designated hospitals.**

# Determination of parameters

# In the models calibration and simulation, the initial values of the first stage SEIAR model is determined as follows:

$$S(\text{Dec 7})=9083500,E(\text{Dec 7})=0,I(\text{Dec 7})=1,A(\text{Dec 7})=0R(\text{Dec 7})=0,$$

where 9083500 is the total population in Wuhan city ^12^. For the second stage SEIAR- CQFH model, the initial values of S, E, I, A, R (Jan 23) are chosen as the end values of the first stage SEIAR model, and the initial value of C, Q, F, H are fixed as follows:

$$C(\text{Jan 23})=0,Q(\text{Jan 23})=0,F(\text{Jan 23})=0,H(\text{Jan 23})=2613,$$

where 2613 is the number of maximum open beds in designated hospitals.

Most of the parameters in SEIAR and SEIAR-CQFH are determined from references (See Table 4 for detailed description) and seven parameters ($\beta_{0},\beta_{end},r,\alpha_{I},\alpha_{H},\delta_{cq},\theta$) are unknown and estimated by stochastic simulation method and the nonlinear least-square method on the basis of newly confirmed, cumulative confirmed and cumulative COVID-19 death cases in Wuhan from Jan 23 to March 18. To start with, we generated 1000 samples of newly confirmed, cumulative confirmed and cumulative COVID-19 death cases from a Poisson process as the counting process was a Poisson process. Secondly, NLES method was used to fit SEIAR and SEIAR-CQFH models and consequently we obtained 1000 groups of values for these estimated values ($\beta_{0},\beta_{end},r,\alpha_{I},\alpha_{H},\delta_{cq},\theta$). All the seven parameters are significant from the results.

# References

1. National Health Commission of the People’s Republic of China. COVID-19.

<http://www.nhc.gov.cn/.> (Date accessed: April 14, 2020).

1. World Health Organization (WHO). Coronavirus disease (COVID-19) Pandemic.

<https://www.who.int/emergencies/diseases/>novel-coronavirus-2019. (Date accessed: April 14, 2020).

1. Wuhan Municipal Health Commission. COVID-19.

<http://wjw.wuhan.gov.cn/.> (Date accessed: April 14, 2020).

1. Chen S, Zhang Z, Yang J, et al. Fangcang shelter hospitals: a novel concept for responding to public health emergencies. *The Lancet,* 2020; **359**: 1305–1314. ­­
2. Sina. Physicians supporting Hubei.

http://blog.sina.com.cn/s/blog_45bb8ce70102yory.html. (Date accessed: April 14, 2020).

1. Zhu N, Zhang D, Wang W, et al. A novel coronavirus from patients with pneumonia in China, 2019. *N Engl J Med*, 2020. **382**: 727–733.
2. Kermack W, McKendrick A. A contribution to the mathematical theory of epidemics. *Proceedings of the royal society of london. Series A, Containing papers of a mathematical and physical character,* 1927; **115(772)**: 700-721.
3. Brauer F, Castillo-Chavez C. Mathematical models in population biology and epidemiology. *New York: Springer*, 2012.
4. Special Expert Group for Control of the Epidemic of Novel Coronavirus Pneumonia of the Chinese Preventive Medicine Association. An update on the epidemiological characteristics of novel coronavirus pneumonia COVID-19. *Chinese Journal of Epidemiology*, 2020; **41(2)**: 139–144.
5. Qiu J. Covert coronavirus infections could be seeding new outbreaks. *Nature*, 2020.
6. Wuhan Municipal Health Commission. Wuhan centralized treatment and isolation of "four categories of personnel".

<http://wjw.wuhan.gov.cn/ztzl_28/fk/fkdt/202004/t20200430_1197006.shtml.> (Date accessed: April 14, 2020).

1. Wuhan Bureau of Statistics. Population.

<http://tjj.wuhan.gov.cn/.> (Date accessed: April 14, 2020).
